# Current landscape of personalized clinical treatments for triple-negative breast cancer

**DOI:** 10.3389/fphar.2022.977660

**Published:** 2022-09-16

**Authors:** Jun Zhang, Yu Xia, Xiaomei Zhou, Honghao Yu, Yufang Tan, Yaying Du, Qi Zhang, Yiping Wu

**Affiliations:** ^1^ Department of Thyroid and Breast Surgery, Shenzhen Qianhai Shekou Free Trade Zone Hospital, Shenzhen, China; ^2^ Department of Gynecological Oncology, Tongji Hospital, Tongji Medical College, Huazhong University of Science and Technology, Wuhan, China; ^3^ Department of Plastic and Cosmetic Surgery, Tongji Hospital, Tongji Medical College, Huazhong University of Science and Technology, Wuhan, China; ^4^ Department of Thyroid and Breast Surgery, Tongji Hospital, Tongji Medical College, Huazhong University of Science and Technology, Wuhan, China

**Keywords:** triple-negative breast cancer, metastasis, biomarkers, targeted therapy, immune therapy

## Abstract

Triple-negative breast cancer (TNBC) is a highly malignant subtype of breast cancer (BC) with vicious behaviors. TNBC is usually associated with relatively poor clinical outcomes, earlier recurrence, and high propensity for visceral metastases than other BC types. TNBC has been increasingly recognized to constitute a very molecular heterogeneous subtype, which may offer additional therapeutic opportunities due to newly discovered cancer-causing drivers and targets. At present, there are multiple novel targeted therapeutic drugs in preclinical researches, clinical trial designs, and clinical practices, such as platinum drugs, poly ADP-ribose polymerase (PARP) inhibitors, immunocheckpoint inhibitors, androgen receptor inhibitors as well as PI3K/AKT/mTOR targeted inhibitors. These personalized, single, or combinational therapies based on molecular heterogeneity are currently showing positive results. The scope of this review is to highlight the latest knowledge about these potential TNBC therapeutic drugs, which will provide comprehensive insights into the personalized therapeutic strategies and options for combating TNBC.

## 1 Introduction

Breast cancer (BC) is a common cancer and is one of the leading causes of cancer-related morbidity and death among women worldwide (Sung et al., 2021). Triple-negative breast cancer (TNBC) is immunohistochemically defined by the lack of expression of estrogen receptor (ER) and progesterone receptor (PR) and human epidermal growth factor receptor 2 (HER2). TNBC is a highly complex and malignant subtype of BC, representing 15–20% of BC (Perou et al., 2000). TNBC usually manifests as the form of high-grade invasive ductal carcinoma and is characterized by a high recurrence rate, often with distant metastases and shorter overall survival (OS) compared to other major BC subtypes (Walsh et al., 2020).

At present, surgery, radiotherapy, and chemotherapy alone or in intriguing combinations, are the main choice for TNBC therapy (Borri and Granaglia, 2021). However, several factors have weakened the therapeutic efficacy of TNBC, including the high propensity to other organ metastases and molecular heterogeneity of TNBC (Li et al., 2021). The FDA approved adjuvants and neoadjuvant regimens, including antimetabolites, taxanes, and Anthracycline, have shown some initial benefit in early TNBC cases, but poor prognosis in late TNBC (Slade, 2020a). Furthermore, unlike hormone therapy and HER2-guided therapy, which produce considerable positive results in hormone receptor (HR)-positive and HER2-positive BC, readily targeted drugs are absent to treat TNBCs compared to other molecular subtypes of BC.

**FIGURE 1:**
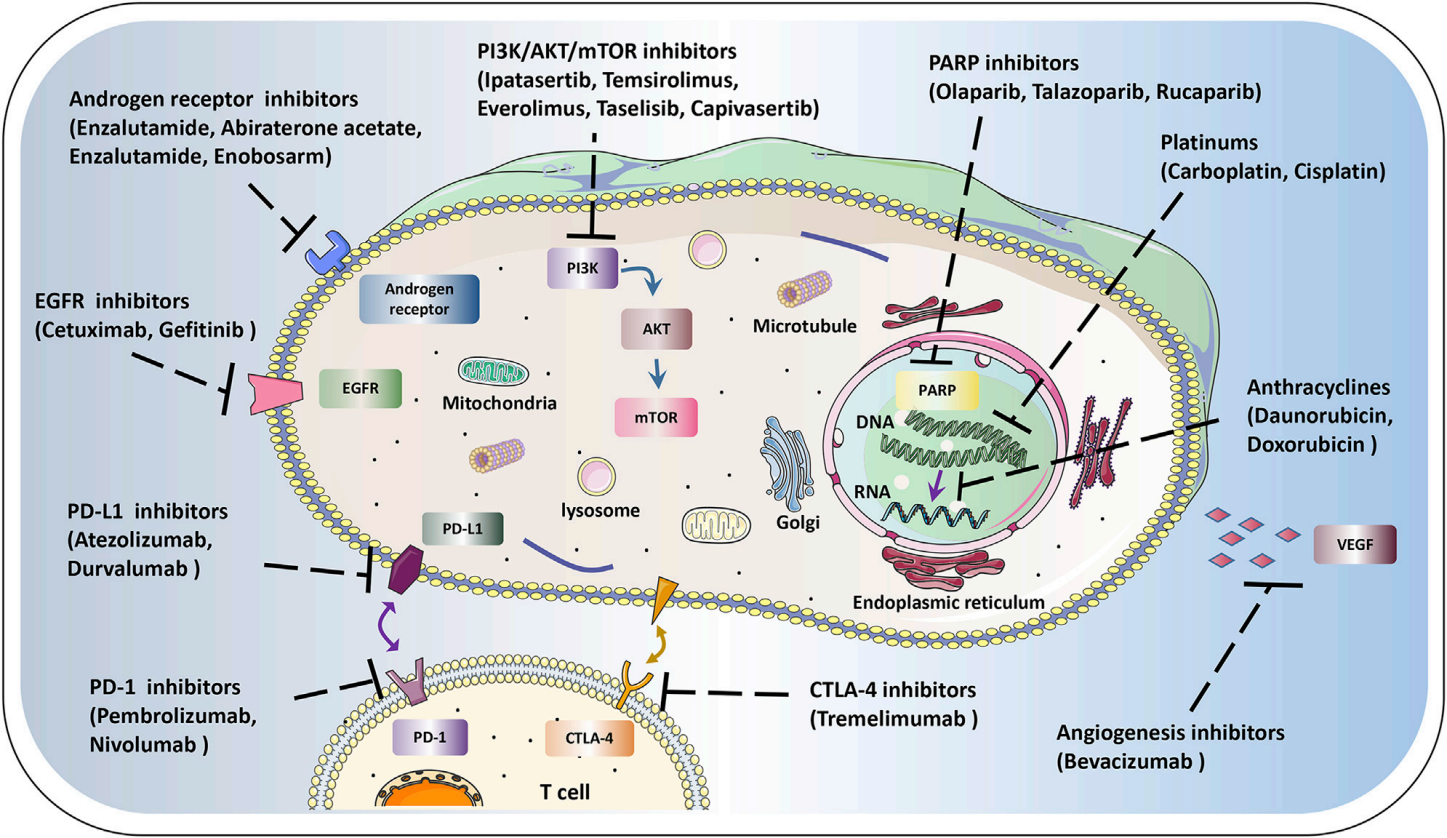
Schematic diagram of therapeutic targets in tumor cells. The current landscape of personalized clinical treatments for TNBC, mainly include anthracyclines platinums, PARP inhibitors, AR inhibitors, ICIs, PI3K/AKT/mTOR targeted inhibitors, EGFR and VEGF inhibitors, etc. Using these novel single or combination therapies will benefit the prognosis of TNBC.

Currently, TNBC is distinguishingly recognized as a very heterogeneous mass with potential therapeutic potential due to newly elucidated carcinogens and targets. Importantly, with the discovery and development of novel treatments, represented by the platinum, poly ADP-ribose polymerase (PARP) inhibitors, immune checkpoint inhibitors (ICIs), androgen receptor (AR) inhibitors, and phosphoinositide-3 kinase (PI3K)/AKT/mTOR targeted inhibitors, the therapeutic options for TNBC are increasing. Translational studies in TNBC have focused on subsets defined by defects in homologous recombination repair, immune cell infiltration, over-activated PI3K pathway, and expression of AR. This article highlights the current landscape of personalized clinical treatments for TNBC therapy (Figure 1). The deep understanding of these novel drugs will provide insight into the therapeutic prospects and possibly redefine the TNBC terminology for combating TNBC.

### 2 Molecular heterogeneity of triple-negative breast cancer

TNBC is the subtype with the worst prognosis due to its very high tumor heterogeneity, drug resistance, and the long-term lack of effective treatment other than chemotherapy (Schmid et al., 2020a). In terms of histological classification, about 95% of TNBC cases are histologically defined as non-specific invasive BC or invasive ductal carcinoma accompanied by no specific histological features (Weigelt and Reis-Filho, 2009). Compared with other BC subtypes, TNBC is more often related to genetic conditions. The histopathological features of TNBC may be caused by different genes and proteins, which provides a theoretical basis for the different pathological features of TNBC at different stages and different levels (Weigelt and Reis-Filho, 2009).

In the early stage, the transcriptome analyses of BC using microarrays were performed to categorize the tumors into five intrinsic subtypes: luminal-A, luminal-B, HER2-enriched, basal-like, and a normal breast-like group (Perou et al., 2000). Although all inherent subtypes can be detected in immunohistochemically defined triple-negative disease, basal-like neoplasms show the greatest degree of overlap with TNBC. Approximately 50% and 75% of TNBC possess a basal phenotype, and approximately 80% of basal-like tumors are ER-negative/HER2-negative (Garrido-Castro et al., 2019). To better demonstrate TNBC-specific tumor heterogeneity, Lehmann et al. determined six TNBC subtypes with a distinct gene expression, including two basal-like subtypes (BL1 and BL2), an immunomodulatory subtype (IM), a mesenchymal subtype (M), a mesenchymal stem-like subtype (MSL), and an intraluminal androgen receptor (LAR) subtype (Lehmann et al., 2011). These types possess distinct gene expression signatures and distinct clinical behaviors. Specifically, there was relatively high expression of cell cycle and DNA damage reaction genes in BL1 and BL2 isoforms. The M and MSL types are characterized by enhanced expression of genes of the epithelial-mesenchymal transition (EMT) and growth-related pathways. The LAR subtype comprises patients with reduced relapse-free survival (RFS) and is characterized by AR signaling (Lehmann et al., 2011).

Besides, by using RNA profiling, Burstein et al. also confirmed 4 stable, clinically relevant TNBC subtypes: LAR, mesenchymal (MES), basal-like immunosuppressed (BLIS), and basal-like immune-activated (BLIA) (Burstein et al., 2015). In addition, these newly described subtypes demonstrate biological diversity, activate diverse molecular pathways, have specific DNA copy number variants (CNVs), and display varied clinical results. “Claudin-low” (CL) tumors are another intrinsic subtype (Prat et al., 2010). Clinically, CL tumors are typified by a lack of expression of luminal differentiation markers, a high degree of enrichment for EMT markers, immunoreactive genes, and cancer stem cell-like signatures. Notably, the majority of CL type is poor prognoses. According to genomic, and transcriptomic information of TNBC patients, Shao et al. divided the TNBC groups into 4 subtypes, including LAR, IM, MES, and BLIS (Jiang et al., 2019). Among them, LAR subtype presents a relatively increased frequency of ERBB2 somatic mutations, less frequency of mutational signature 3, and more CDKN2A loss. Each TNBC subtype contains specific potential therapeutic targets. Therefore, the comprehensive profile of TNBCs will valuable reference for precise tumor treatment.

### 3 Novel trials for clinical triple-negative breast cancer treatment

#### 3.1 Platinums

Platinum-based chemotherapy (PBC) has been extensively studied in TNBC over the past decade, showing potential as an important primary treatment for metastatic triple-negative breast cancer (mTNBC) (Dent et al., 2021). Among the multiple platinum-based drug administrations, the cisplatin-based treatment program performed best (Dieci et al., 2019). These platinum-based agents can generate intra-chain and inter-chain double-stranded DNA crosslinks, which inhibit replication fork formation and mediate apoptosis of tumor cells (Zhou et al., 2020). Sensitivity to PBC might attribute to the damage and dysfunction by DNA cross-linking in tumor cells.

BRCA mutation status is a valuable and reliable diagnostic marker in TNBC (Sporikova et al., 2018). The therapeutic effect of platinum drugs (including carboplatin and cisplatin) in TNBC is significantly associated with BRCA mutations (Reis-Filho and Tutt, 2007). Advanced TNBC patients benefit from are prone to get benefit from BRCA1/2 mutation profile for providing platinum selection (Tutt et al., 2018). Single cisplatin or carboplatin has a significant objective response rate (ORR) in TNBC patients with BRCA1 mutation, but the role of platinum in non-BRCA-mutated mTNBC needs to be verified (Ghebeh et al., 2021). In the trial of TBCRC 030, Mayer et al. noted that homologous recombination defect (HRD) was not predictive of pathological response to preoperative cisplatin or paclitaxel in TNBC patients (Mayer et al., 2020). Wang et al. enrolled in a trial to compare the efficacy of PBC and non-PBC regimens in treating advanced TNBC (Wang et al., 2020). The median progression-free survival (PFS) in patients with or without deleterious mutations was 14.9 and 5.3 months, and the median OS was 26.5 and 15.5 months. In addition, because PBC was more functional in patients with harmful mutations, genetic testing in patients with advanced TNBC might yield better results.

Current ongoing trials evaluating the effectiveness of PBC versus other chemotherapy regimens will contribute to better management of TNBC. Chen et al. compared the effectiveness of PBC and non-PBC in advanced TNBC patients in four cancer centers in China. Notably, PBC doublets exhibited superior efficacy and tolerable toxicity compared with non-PBC doublets in the first-line treatment for mTNBC patients (Xu et al., 2020). This approach and conclusion were similar to the previous Canadian multicenter study of Villarreal-Garz et al. group, which confirmed that PBC-treated TNBC patients exhibited a more positive OS compared with conventionally managed TNBC patients with non-platinum chemotherapy (Sirohi et al., 2008). In the phase I/II MBC-10 trial, Rinnerthaler et al. assessed the efficacy of ixazomib, a proteasome inhibitor, combined with carboplatin, confirming that this combination was effective in treating patients with advanced TNBC (Rinnerthaler et al., 2018).

Neoadjuvant chemotherapy (NAC), which refers to the use of chemotherapeutic drugs before surgery, is a non-elimination disease modality but can improve the outcome of surgical procedures and is mainly applicable to patients with malignant tumors that have not developed distant metastases and are locally progressive (Wang and Mao, 2020). In a retrospective study, Elaine et al. demonstrated that the pathological complete response (pCR) rate was able to predicate the therapeutic ends and supported the utilization of carboplatin in NAC for TNBC (Walsh et al., 2019). Dieci et al. proposed a clinical protocol to include platinum in anthracycline/taxane-based NAC therapy for TNBC (Dieci et al., 2019). The incidence of pCR was higher in the PBC group than in the control group. Du et al. enrolled a non-inferior randomized phase 2 trial to confirm that carboplatin with taxanes was of capability in reliable adjuvant chemotherapy for TNBC patients in early-stage who did not withstand the chemotherapy with anthracycline (Du et al., 2020). Nevertheless, in the cohort of patients with basal subtype TNBC and residual invasive disease, platinum-based drugs did not improve the prognosis and exhibited a more toxic feature than capecitabine, accompanied by lower anticipated invasive disease-free survival (iDFS) (Mayer et al., 2021). This finding suggested that platinums were not appropriate or forbidden as the adjuvant agents in this population, highlighting better treatment strategies for this high-risk group. Furthermore, since incomplete pCR response in patients after NAC results in poor prognosis, effective characterization of the molecular profile to facilitate pCR prediction is critical. Ademuyiwa et al. reported a stable pCR rate of 45.7% in TNBC patients treated with neoadjuvant docetaxel and carboplatin regimen, and posed that tumor-associated mutation of epidermal growth factor receptor (EGFR), RB1, RAD51AP2, SDK2 and so on, together with immune-related gene (IRGs), might differentiate TNBC patients who would obtain pCR in this regimen (Slade, 2020b).

Platinum-based adjuvant chemotherapy is still controversial in TNBC patients regardless of whether the BRCA1 and BRCA2 (BRCA1/2) germline variants are related to treatment with platinum. Yu et al. designed a phase 3 randomized clinical trial comparing paclitaxel plus carboplatin (PCb) at 6 cycles with standard-dose regiments of cyclophosphamide, epirubicin, fluorouracil, and docetaxel at 3 cycles (Yu et al., 2020). These findings indicated that the PCb regimen was a viable selection for adjuvant chemotherapy in those cohorts with operable TNBC. Now, the subgroups sensitive to PCb need to be further studied. The addition of carboplatin to anthracycline, cyclophosphamide, and taxane regimen was associated with improved complete pathologic response (pCR), whereas patients who did not achieve pCR had a high risk of recurrence (Lee et al., 2020). Another Phase II trial examined the outcomes of the non-anthracycline plus carboplatin and nab-paclitaxel in treating early-stage TNBC (Bianchini et al., 2022). The results showed well-tolerated, highly effective effects and a pCR of 48%. Besides, they found that the GeparSixto immune profile was associated with a higher pCR, with positive implications for downgrading to NAC.

Despite these encouraging clinical data of PBC in TNBC, it is worth noting that there are some challenges in incorporating platinums into standard clinical practice. First of all, as not everyone has an exact response to treatment, predictive biomarkers are still needed to stratify patients with TNBC to avoid the adverse toxicity of these drugs. Besides, to date, there is not enough evidence have been powered to prove the long-term survival outcomes. Besides, the toxicities, dosing and regimen strategies are important clinical questions to ponder. It is very important for platinum to cooperate with other chemotherapeutic medicines to improve pCR and survival. And, Overall, platinum chemotherapy is promising but still deserves deeper and further exploration.

### 3.2 Poly ADP-ribose polymerase inhibitors

The inherent genomic instability of TNBC is closely connected to DNA repair defects. This is identifiable by mutational signature analysis and might be targetable with poly (ADP-ribose) polymerase (PARP) inhibitors (Slade, 2020c). BRCAness is an index with great predictive value of curative effect of platinum and PARP inhibitors, which are validated to preclinical and clinical activity (Pilié et al., 2019). The PPAR inhibitors olaparib and talazoparib, have been allowed for metastatic BC therapy, including TNBC cohort with germline mutations. It also has been demonstrated that PARP suppression reinforces the functions of ionizing radiation agents, DNA-methylating compounds, and PBC (Hastak et al., 2010).

Olaparib could enhance the anti-tumor immune by governing T-cell infiltration mediated through STING/TBK1/IRF3 pathway activation (Pantelidou et al., 2019). It elucidates another mechanism of PARP inhibitors and provides the basic principle of combining PARP inhibition with immunotherapy to treat TNBC. In phase I/II trial in Japanese patients with advanced or mTNBC, the combination treatment of olaparib with eribulin exhibited effective anti-tumor ability, with caution in the presence of febrile neutropenia (Voorwerk et al., 2019). GeparOLA (NCT02789332) studied the efficacy of olaparib in combination with paclitaxel in the treatment of BC (Fasching et al., 2021). PCR rates of olaparib and carboplatin in TNBC patients were 56.0% and 59.3%, respectively. In a phase 2 window clinical trial, RIO trial (EudraCT 2014–003319-12), exploration of 43 patients with untreated TNBC, PARP inhibitor rucaparib induced expression of interferon response genes and suppressed circulating tumor DNA (ctDNA) in homologous recombination-deficient TNBC (Chopra et al., 2020).

During the phase II I-SPY2 trial, the united therapy of durvalumab and olaparib was given to the standard NAC therapy with paclitaxel, denoted as DOP (Pusztai et al., 2021). The pCR rates with 27%–47% were markedly improved in TNBC patients with DOP, than those cohorts with paclitaxel standard. Eikesdal et al. enrolled phase II PETREMAC trial that TNBC patients with solid tumors larger than 2 cm received olaparib for 10 weeks prior to chemotherapy (Oliveira et al., 2019). Olaparib achieved an objective response in 18 of 32 patients (56.3%) with mild side effects and did not affect the tolerability of subsequent chemotherapy. Furthermore, HRD could predict the olaparib efficacy and response to olaparib over germinal BRCA1/2 and gPALB2 mutations. Therefore, the germline BRCA1/2 mutations, HRD, as well as homozygous BRCA1 promoter methylation are potentially useful indicators for evaluating the stratification of TNBC patients who would reap the benefits of PARP inhibitor olaparib/eribulin co-administration (Kawachi et al., 2020).

### 3.3 Androgen receptor

AR is another area of great interest and is emerging as an important factor in the pathogenesis of BC. AR is expressed in BC tissues and normal tissues to varying degrees, and AR expression of BC tissues shows significant differences in different stages, pathological types, and malignant degrees. (Finn et al., 2020). About 10–15% distinct subgroups of TNBC with AR present more benign processes, which may be responsive to AR blockade. Besides, the genome of these tumors is featured by an enriched rate of PIK3CA-activated mutations. The first proof-of-concept trial confirmed the efficacy of the AR antagonist bicalutamide in patients with advanced AR-positive TNBC (Gucalp et al., 2013). Since then, evidence of clinical efficacy further supports the therapies of other next-generation AR-targeted agents such as enzalutamide, abiraterone acetate, and enzalutamide. Abiraterone acetate plus prednisone treatment was beneficial for some patients with AR-positive locally advanced or mTNBC (Bonnefoi et al., 2016). Biological insight into AR-positive TNBC has positive significance for signaling pathway cross-talk, TNBC subtype classification, early tumor detection, and synergistic treatment.

Preclinical data have demonstrated that AR-positive TNBC cells respond effectively to AR antagonists. Enobosarm, also known as GTx-024 or ostarine, is a first-in-class, nonsteroidal selective androgen receptor modulator (SARM) being developed for diverse indications in medical oncology, including AR-positive BC (Dalton et al., 2011). Yuan et al. explored the potency and biosafety of enobosarm and pembrolizumab combination in AR + mTNBC patients (Yuan et al., 2021). The enobosarm plus pembrolizumab was well tolerated, in heavily pre-treated AR TNBC without pre-selected PD-L1. Therefore, it is necessary to further conduct clinical trials on the combination of AR-antagonistic treatment with immunosuppressant for AR + TNBC. A phase II study in 2018 was performed to figure out the benefit of enzalutamide in patients with locally advanced or metastatic AR-positive TNBC (Traina et al., 2018). The clinical benefit rate (CBR) was 25% and 33%, median PFS was 2.9 months and 3.3 months, respectively in the intention to treat (ITT) population and the evaluable subgroup. Thus, enzalutamide exhibited a positive profile in clinical activity and tolerance in advanced AR-positive TNBC patients. In the multi-institutional phase Ib/II study TBCRC032, patients with stage II TNBC were randomized to be treated with enzalutamide alone or plus taselisib (Lehmann et al., 2020). In this study, Lehmann et al. validated the treatment effectiveness of enzalutamide combined plus taselisib in AR + TNBC with a better CBR (35.7%) and PFS (3.4 months). Moreover, genomic analyses revealed that only AR protein expression was not sufficient for identifying patients, confirming the necessity of identifying tumor LAR subtypes or AR splice variants in AR antagonist therapy.

However, the prognostic characteristics of AR expression in TNBC patients continue to be debated. A meta-analysis study explored the relationship between AR expression and survival outcomes and found that there was no correlation (Xu et al., 2020). It seems difficult to identify and stratify patients who will benefit from AR-targeted therapy. AR-based prognostic and predictive value and the related biomarker identification improve response rates would be encouraging.

### 3.4 PI3K/AKT/mTOR targeted therapy

Abnormal PI3K/AKT/mTOR is frequently activated in TNBC and is linked with oncogenesis, progress, and chemoresistance. Activation of the PI3K pathway is mainly triggered upon the protein level, commonly accompanied by less dependent on PIK3CA mutations and loss of the negative regulators PTEN (Oliveira et al., 2019). High-frequency alterations in PI3K pathway gene provide a theoretical basis for designing inhibitors targeting PI3K/AKT. In BRCA-proficient TNBC, PI3K inhibition impairs BRCA1/2 expression and DNA homologous recombination, and sensitizes TNBC to PARP inhibition (Ibrahim et al., 2012). Recently, PI3K blockers have displayed several desirable therapeutic effects in patients with stage II-III TNBC with PIK3CA mutations (Gupta et al., 2020).

Ipatasertib is a highly selective AKT kinase small molecule inhibitor currently for treating BC and prostate cancer (Sweeney et al., 2021). In a phase 2 trial (LOTUS), the results supported the AKT-targeted therapy for TNBC. Median PFS in the intention to treat (ITT) population was 6.2 months with ipatasertib versus 4.9 months with placebo. In 48 patients with tumors with low PTEN levels, median PFS was 6.2 months for ipatasertib compared with 3.7 months for placebo (Sweeney et al., 2021). The final result of this program in 2021 revealed that patients with advanced TNBC could benefit from the ipatasertib-paclitaxel combination (Dent et al., 2021). In the PAKT trial of AKT inhibitor capivasertib mTNBC, Schmid et al. verified that adding the capivasertib to the first-line paclitaxel treatment in TNBC could observably prolong PFS and OS for benefiting TNBC patients (Schmid et al., 2020b). The FAIRLANE trial showed a consistent result from LOTUS and PAKT in metastatic TNBC (Oliveira et al., 2019). In FAIRLANE, the addition of ipatasertib to paclitaxel NAT in early TNBC did not pose a clinically significant enhancement in pCR rates, but ipatasertib had a more pronounced antitumor effect in patients with biomarker selection.

To ascertain whether the prognosis is affected by tumor subtype, Basho et al. evaluated the results of therapy in patients with mesenchymal breast cancer (MpBC) versus non-MpBC, undergoing mTOR inhibition (temsirolimus or everolimus) with liposomal doxorubicin and bevacizumab (Basho et al., 2018). The results demonstrated that patients with advanced MpBC showed better long-term outcomes, which indicated that metaplasia histology might predict the efficacy of agent targeting the PI3K/Akt/mTOR pathway. In multi-institutional phase Ib/II study TBCRC032, the combination of AR-antagonist enzalutamide and PI3K inhibitor taselisib could promote CBR (Lehmann et al., 2020). In the SAFIR02 trial (NCT02299999), Mosele et al. selected a total of 649 patients with metastatic BC with available mutational profiles for outcome analysis, interestingly showing that TNBC recipients with PIK3CA mutation exhibited superior OS. This might be due to the accumulation of PIK3CA mutations in luminal BC and the reduction of HR performance during metastasis (Mosele et al., 2020). Anand et al. evaluated the efficacy of targeted therapy following standard anthracycline and taxane NAC, which showed that everolimus combined with cisplatin was effective for TNBC patients with residual lesions following standard NAC (Anand et al., 2021).

However, in a randomized phase II neoadjuvant study, the adjuvant treatment with mTOR inhibitor everolimus did not increase response rates and was associated with more adverse events, in stage II/III patients with TNBC who received weekly preoperative weekly cisplatin, paclitaxel, and daily everolimus or placebo before surgery (Anand et al., 2021). Therefore, how to reconcile the dose of the three drugs, synergistically exert the function of inhibitors, and more effectively “attack” TNBC is a challenge. In addition, drugs targeting distinct PI3K/AKT/mTOR components and homologous molecules (e.g., MAPK) are in urgent development. In this evolving field, in addition to the effects and application range of these drugs, it is also necessary to consider synergistic treatment with the other targeted TNBC drugs.

### 3.5 Immune therapy

The host immune system is an important orchestrator in reshaping the response to TNBC treatment and prognosis. High levels of intratumoral tumor-infiltrating lymphocytes (TILs) in TNBC are a potential biomarker for indicating a more favorable survival outcome and response to immunotherapy in TNBC (Lotfinejad et al., 2020). In addition, immunosuppressive programmed cell death ligand 1 (PD-L1) is expressed in 20% of TNBC (Dieci et al., 2015). TNBC contains high levels of TILs and CD8^+^ lymphocytes, as well as expressing PD-L1, which seems to have greater clinical benefits (Ghebeh et al., 2021). Immunomodulatory antibody-based strategies are emerging as promising approaches to bring breakthroughs to TNBC. Several ICIs, including PD-L1-targeted (atezolizumab, and durvalumab), PD-1-targeted (pembrolizumab, nivolumab, and camrelizumab), CTLA-4-targeted (tremelimumab) antibodies, are currently being investigated either as monotherapy or co-therapy for TNBC in many immunotherapy trials.

ICIs have the capabilities to dramatically enlarge the treatment efficacy in TNBC. Besides, other hopeful immunotherapy methods of TNBC, like anti-protease cathepsin D, or EGFR therapy, personalized vaccination, adoptive cell therapy, Immunotherapy combination, are also under comprehensive and deep investigation (Ashraf et al., 2019) (Jia et al., 2017). Other ICIs for single agent treatment, or in association with pharmacotherapy for advanced TNBC, and for early stage disease, are emerging and ongoing areas of research. This will be a key direction for future clinical trials to characterize highly predictable biomarkers of response, as well as strategies for personalized immunotherapy in the treatment of TNBC.

#### 3.5.1 PD-L1-targeted therapy

Atezolizumab is a humanized, PD-L1-targeted monoclonal antibody that is well tolerated and clinically improves the anti-tumor activity in multiple cancer types (Finn et al., 2020). Atezolizumab coupled with nab-paclitaxel is representative of a promising novel first-line care standard for those with PD-L1-positive mTNBC. In a phase I study, the women with mTNBC were treated with single-agent atezolizumab every 3 weeks (Emens et al., 2019). This study preliminarily verified the safety and clinical activity of atezolizumab with a 10% ORR and an OS of 17.6 months in the entire cohort. To extend the optimal anti-tumor activity, combinations with standard chemotherapy strategies are being investigated to synergistically elicit the death of immune tumor cells. Adams et al. further conducted a phase Ib trial of atezolizumab in the context of nab-paclitaxel the cohort of mTNBC (Adams et al., 2019a). The ORR was 39.4% and the median PFS and OS were 5.5 months and 14.7 months, respectively. It demonstrated that this combination mTNBC was reliable and offered a manageable safety profile. Next, Schmid et al. reported a second interim OS analysis of the phase 3 IMpassion130 study, which indicated a valuable OS benefit of this regimen in patients with PD-L1 immune cell-positive BC by receiving atzolizumab plus nab-paclitaxel in cohort with unresectable, localized advanced, or mTNBC (Schmid et al., 2020a). It was also intriguing that early TNBC patients receiving neoadjuvant therapy combining azolizumab with sequential nab-paclitaxel and anthracycline-based treatment, presented markedly higher pCR and excellent biosafety characteristics (Mittendorf et al., 2020).

In GeparNuevo trial, durvalumab was added to standard NAC therapy in early TNBC patients (Loibl et al., 2019). The results deciphered that the joining of durvalumab to anthracycline/paclitaxel-based NAC strategy might improve pCR rates to some extent, particularly in patients treated with durvalumab alone prior to chemotherapy initiation. Meanwhile, the cohort of this randomized phase II placebo-controlled study was too small to give very definitive evidence. The usage of durvalumab has some positive implications in patients with higher tumor load. Ghebeh’s study investigated the efficacy of durvalumab and paclitaxel in combination for TNBC treatment (Ghebeh et al., 2021). The results showed that patients who underwent at minimum one period of combined therapy demonstrated a positive safety profile, indicating the promise and window for further use of the therapy. In addition, Ahmed et al. suggested that higher levels of PD-L1 expression in tumor cells, stromal immune cells, and co-localization of CD68-positive cells were strongly linked to superior pCR of NAC durvalumab in TNBC group (Ahmed et al., 2020).

#### 3.5.2 PD-1-targeted therapy

Notably, the combination of ICI antibodies and other tumor-targeting drugs can produce significant synergistic effects. To explore the optimal setting and potential mechanisms of anti-angiogenesis inhibitors and anti-PD-1 therapy, Liu et al. preliminarily revealed dose-dependent synergistic effects of this combination at animal and TNBC patient levels and showed that increased osteopontin (OPN) and TGF-β expressions were positively correlated with favorable therapeutic effect (Li et al., 2019). In their subsequent clinical trial (NCT03394287), the ORR of camrelizumab and apatinib combination therapy was significantly higher than that of camrelizumab or apatinib alone (Liu et al., 2020). Therefore, camrelizumab in combination with apatinib displayed good therapeutic function and controllable results in the advanced TNBC cohort. Within this therapy, several responsive biomarkers could predict better ORR, including higher baseline TIL and tumor-infiltrating CD8^+^ T cells, and enhanced plasma TIM-3/CD152, as well as lower baseline plasma HGF/IL-8 and decreased plasma IL-8 level (Liu et al., 2021).

Pembrolizumab is a PD-1 inhibitor currently being used in clinical trials that possesses positive efficacy in solid tumors, represented by recurrent/metastatic cervical cancer and advanced melanoma (De Felice et al., 2021) (Slade, 2020a). The phase Ib KEYNOTE-173 aimed to evaluate the security and preliminary efficacy of NAC in combination with pembrolizumab utilized in the cohort of high-risk, early-stage non-mTNBC (Dent et al., 2021). The pCR rates for all cohorts ranged from 49% to 71%, indicating that this treatment, NAC plus pembrolizumab, had definite antitumor activity and was associated with low side effect symptoms.

The efficacy of therapeutic strategies of PD-1 blockade alone is insufficient in metastatic TNBC. This is because many patients with metastatic tumors exhibit resistance to therapy. Moreover, although some patients can benefit from PD-1/PD-L1 blockade therapy in the setting of PD-L1-positive TME, overall the majority of patients benefit less (Adams et al., 2019b). Therefore, how to effectively improve or amplify the therapeutic effect of PD-1/PD-L1 blockade is an important obstacle to be solved urgently. In the TONIC trial, Voorwerk et al. pointed out that short-term doxorubicin and cisplatin might trigger a more improved TME, manifested by up-regulation of PD-1, PD-L1, and IRG expression in T cell cytotoxicity signal pathway (Voorwerk et al., 2019). Therefore, the combination of chemotherapy and PD-1 blockade makes TNBC tumor cells more sensitive to PD-1 blockade, and most patients in the treatment group showed effective ORR (Table 1).

**TABLE 1:** Current PD-L1/PD-1 inhibior trials in TNBC patients.

Agent catalogues	Trials	Patients	Therapeutic regimens	Key results	Adverse events
Atezolizumab (PD-L1 inhibitor) (Emens et al., 2019)	PCD4989g, phase Ⅰ trial (NCT02447003)	116 women with mTNBC	Atezolizumab intravenously at 15 or 20 mg/kg, or at a 1,200 mg, q3 wks	ORRs were 24% in first-line and 6% in second-line or greater patients; mPFS were 1.4 (95% CI 1.3–1.6) months by RECIST and 1.9 (95% CI 1.4–2.5) months by irRC; OS 17.6 months (95% CI 10.2 months-not estimable)	The most frequent TRAEs were pyrexia 16%, fatigue 13%, and nausea 11%, followed by diarrhea 10%, asthenia 10%, and pruritus 10%
Atezolizumab (PD-L1 inhibitor) + nab-PTX (Adams et al., 2019a)	GP28328, phase 1b study (NCT01633970)	33 women patients with stage IV or locally recurrent TNBC; no more than 2 prior systemic cytotoxic regimens or not received a taxane within 6 months prior to enrollment	Patients in the safety cohort received atezolizumab, 800 mg, on days 1 and 15 of each cycle q2 wks + nab-PTX 125 mg/m^2^ on days 1, 8, and 15 of each cycle; Patients in the serial biopsy cohort received nab-PTX alone on days 1 and 8 of cycle 1, followed by concurrent nab-PTX and atezolizumab 800 mg, starting on day 15	mPFS 5.5 months (95% CI 5.1–7.7); OS 14.7 months (95% CI 10.1-not estimable)	100% patients experienced at least 1 treatment-related AEs, 73% patients experienced grade 3/4 EVs, and 21% patients had grade 3/4 AESI
Atezolizumab (PD-L1 inhibitor) + nab-PTX (Schmid et al., 2020a)	IMpassion130, phase Ⅲ study (NCT02425891)	902 patients aged 18 years or older, with previously untreated mTNBC	Atezolizumab or placebo 840 mg intravenously on day 1 and day 15 of every 28 days cycle and nab-PTX 100 mg/m^2^ intravenously on days 1, 8, and 15	mOS were 21.0 months (95% CI 19.0–22.6) with atezolizumab and 18.7 months (16.9–20.3) with placebo	The most common grade 3/4 EVs were neutropenia 8% in the atezolizumab group vs. 8% in the placebo group, peripheral neuropathyb6%vs. 3%
Atezolizumab (PD-L1 inhibitor) + nab-PTX + ADM + CP (Mittendorf et al., 2020)	IMpassion031, phase Ⅲ study (NCT03197935)	333 patients aged 18 years or older; clinical stage cT2-T4 and cN0-cN3 histologically documented TNBC; no previous systemic therapy with anthracyclines or taxanes for any malignancy	Atezolizumab or placebo 840 mg q2 wks + nab-PTX 125 mg/m^2^ weekly for 12 w, followed by intravenous atezolizumab or placebo 840 mg q2 wks + ADM 60 mg/m^2^ + CP at 600 mg/m^2^ q2 wks	pCR was 95 (58%, 95% CI 50–65) patients in the atezolizumab + chemotherapy group and 69 (41%,95% CI 34–49) patients in the placebo + chemotherapy group (rate difference 17%, 95% CI 6–27; one-sided *p* = 0.0044)	In the neoadjuvant phase, grade 3–4 AE were balanced and treatment-related serious EVs occurred in 23) and 16% patients, with one patient per group experiencing an unrelated grade 5 EVs
Durva (PD-L1 inhibitor) + nab-PTX (Loibl et al., 2019)	GeparNuevo, phase II study (NCT02685059)	174 patients with previously untreated uni- or bilateral primary, non-metastatic invasive TNBC; with a tumor of at least 2 cm (cT2-cT4a-d)	Durva or placebo 0.75 g for 2 weeks before start of chemotherapy, followed by durva or placebo 1.5 g q4 wks + nab-PTX 125 mg/m^2^ weekly for 12 weeks, followed by durva or placebo 1.5 g q4 wks + EC q2 wks	pCR were 53.4% (95% CI 42.5%–61.4%) in durva group and 44.2% (95% CI 33.5%–55.3% in placebo group, corresponding to OR = 1.45 (95% CI 0.80–2.63)	AEs were not more frequently reported with durva than with placebo, with the exception of thyroid dysfunction, which was more frequently reported on durva
Durva (PD-L1 inhibitor) + nab-PTX (Ghebeh et al., 2021)	Single-arm, phase I/II study, (NCT02628132)	14 mTNBC patients with no immune-related diseases, or symptomatic/uncontrolled brain metastasis	A single cycle of PTX alone followed by 5 cycles of a combination of weekly PTX 80 mg/m^2^ on days 1, 8, and 15 and durva 750 mg as fixed-dose	ORR with and without the relapsing were 25% and 36%; mPFS 4.0–5.0 months; OS 20.7 months	Regardless of grade, the most common AEs were headache and peripheral neuropathy, (29%), followed by fatigue and skin rash (21%)
Durva (PD-L1 inhibitor) + nab-PTX + ADM + CP (Ahmed et al., 2020)	Single arm Phase I/II trial, (NCT02489448)	45 patients with newly diagnosed stage I-III TNBC and had not undergone surgical treatment	Durva 10 mg/kg q2 wks for 19 weeks + nab-PTX 100 mg/m^2^ weekly for 12 weeks + ADM 60 mg/m^2^ + CP 600 mg/m^2^, every other week from 13 to 19 weeks	pCR = 18, non-pCR = 27; In patients with pCR, PD-L1 expression was significantly higher in tumor cells, in CD68^+^ cells and in the stroma compared with patients non-pCR	N/A
Camrelizumab (PD-1 inhibitor) + apatinib (VEGFR2-targeting agents) (Li et al., 2019)	phase II trial, (NCT03394287)	12 women ages 18- to 70-years-old with metastatic or unresectable recurrent TNBC	Apatinib 250 mg/d, + camrelizumab 200 mg q2 wks	mPFS 3.7 months (95% CI, 1.7-not reached); In patients with advanced TNBC, combined treatment with low-dose anti-VEGFR2 inhibitor and anti-PD-1 demonstrated excellent tolerability and efficacy	Regardless of grade, the most common AEs were elevated AST (66.7%), elevated ALT (58.3%), and hand-foot syndrome (58.3%)
Camrelizumab (PD-1 inhibitor) + apatinib (VEGFR2-targeti ng agents) (Liu et al., 2020)	Two-arm, phase II trial (NCT03394287)	40 female patients (18–70 years) with metastatic or unresectable recurrent TNBC; no history of severe allergic reaction to other monoclonal antibodies	Camrelizumab 200 mg (3 mg/kg for patients < 50 kg) intravenously, q2 wks + apatinib 250 mg oral, continuous dosing (d1-d14) or intermittent dosing (d1-d7)	PFS were 3.7 (95% CI 2.0–6.4) months and 1.9 (95% CI 1.8–3.7) months in the continuous dosing and intermittent dosing cohort	The most common AEs included elevated AST/ALT and hand-foot syndrome
Camrelizumab (PD-1 inhibitor) + apatinib (VEGFR2-targeti ng agents) (Liu et al., 2021)	Two-arms phase II trial (NCT03394287)	28 female patients with metastatic or unresectable recurrent TNBC; no history of severe allergic reaction to other mAbs	Camrelizumab 200 mg (3 mg/kg for patients whose weight was below 50 kg) intravenously, q2 weeks, + apatinib 250 mg oral, continuous dosing (d1-d14) or intermittent dosing (d1-d7)	mPSF in patients with lower baseline plasma levels of HGF or IL-8 were more likely to respond to treatment (*p* = 0.005 or 0.001), and showed a longer mPFS (7.69 months vs. 2.10 months and 8.25 months vs. 1.98 months) and OS (not reached vs. 3.86 months and not reached vs. 4.7 months)	Patients with lower baseline plasma IL-18, or IFN-γ levels were more likely to suffer from gastrointestinal TRAEs; patients with higher baseline plasma VEGF-A or MIP-1β were more likely to have respiratory TRAEs
Pembrolizumab (PD-1 inhibitor) + nab-PTX + CBP AUC5 (Dent et al., 2021)	KEYNOTE-173, phase Ib study (NCT02622074)	60 women patients with previously untreated, high-risk, early-stage, non-metastatic (M0) TNBC (T1c, N1-N2; T2-T4c, N0-N2)	Pembrolizumab 200 mg (cycle 1) then 8 cycles of pembrolizumab + PTX (125 mg/m^2^, 100 mg/m^2^, 80 mg/m^2^) with or without CBP for 12 weeks + ADM and CP for an additional 12 weeks before surgery	The pCR rate across all cohorts was 60% (range 49%–71%) OS rates ranged from 80% to 100% across cohorts (100% for four cohorts); Pre- and on-treatment sTILs were significantly associated with higher pCR rates (*p* = 0.0127, 0.0059, and 0.0085, respectively)	Dose-limiting toxicities occurred in 22 patients, most commonly febrile neutropenia. The most common grade ≥ 3 treatment-related EVs was neutropenia (73%)
Pembrolizumab (PD-1 inhibitor) (Adams et al., 2019b)	KEYNOTE-086, phase II study, cohort B, (NCT02447003)	84 women patients with mTNBC with no prior systemic treatment of metastatic disease and had PD-L1-positive tumors	Pembrolizumab 200 mg intravenously over 30 min q3 weeks for up to 2 years	mPFS 2.1 months (95% CI 2.0–2.2); mOS was 18.0 months (95% CI 12.9–23.0)	53 (63.1%) patients experienced ≥ 1 TRAEs. The most common TRAEs were fatigue (26.2%), nausea (13.1%), and diarrhea (11.9%)
Nivolumab (PD-1 inhibitor),CP + DDP + ADM (Voorwerk et al., 2019)	TONIC trial, phase Ⅱ study (NCT02499367)	67 patients with mTNBC aged 18 or older	Four different induction treatments, consisted of irradiation to a single lesion (3 fractions of 8 Gy within 10 weekdays after randomization), CP (50 mg orally daily for 2 weeks), DDP (40 mg/m^2^ intravenously weekly for 2 weeks) or ADM (15 mg intravenously weekly for 2 weeks)	In the overall cohort, the ORR was 20%. The majority of responses were observed in the DDP (ORR 23%) and ADM (ORR 35%) cohorts	Induction treatment-related AEs of any grade occurred in 19 patients (28%, with 3% grade 3) and immune-related AEs of grades 3–5 occurred in 13 patients (19%)

adverse event (AE); AEs, of special interest (AESI); alanine aminotransferase (ALT); aspartate aminotransferase (AST); carboplatin (CBP); confidence interval (CI); cisplatin (DDP); cyclophosphamide (CP); durvalumab (durva); Eastern Cooperative Oncology Group performance status (ECOG PS); every 4 weeks (q4 wks); doxorubicin (ADM); immune-related response criteria (irRC); median OS (mOS); median progression-free survival (mPFS); metastatic triple-negative breast cancer (mTNBC); monoclonal antibodie (mAb); albumin bound paclitaxel (nab-PTX); objective response rate (ORR); overall survival (OS); pathological complete response (pCR); programmed cell death ligand 1 (PD-L1); progression-free survival (PFS); Response Evaluation Criteria in Solid Tumors (RECIST); stromal tumor-infiltrating lymphocyte levels (sTILs); Treatment-related AEs (TRAEs); triple-negative breast cancer (TNBC); vascular endothelial growth factor receptor (VEGFR); weeks (wks).

### 3.5.3 Antibody-drug conjugates

Antibody-drug conjugates (ADCs) are novel engineered therapeutic agents consisting of a humanized monoclonal antibody targeting a tumor-specific antigen and a loaded cytotoxic drug. Many ADCs have demonstrated impressive performance of high efficacy and safety in a variety of cancers including breast, lung, and hematological malignancies. Some ADCs, including glembatumumab vedotin (CDX-011, GV), sacituzumab govitecan, PF-06647263, and mirvetuximab soravtansine, have becoming increasingly important options in TNBC trials.

GV belongs to a ADC with a glycoprotein NMB-targeting monoclonal antibody conjugated with monomethyl auristatin E (MMAE) (Bardia, 2017). Vahdat et al. conducted 2 breast cancer clinical trials based on Glembatumumab vedotin. They firstly enrolled 42 patients with advanced/metastatic breast cancer, who were treated with standard 3 + 3 dose escalation followed by a phase II extension, and were confirmed by immunohistochemical gpNMB staining of tumor tissue (Bendell et al., 2014). Median PFS was 17.9 weeks for TNBC patients, and 18 weeks for patients with gpNMB + tumors, preliminarily confirming the tolerability of this ADC. Subsequent EMERGE clinical trial results showed that glembatumumab vedotin had a good and controlled safety feature, and that its activity was possibly intensified in patients with TNBC and/or gpNMB tumor expression (Yardley et al., 2015).

Sacituzumab govitecan is a FDA-approved ADC for pretreated mTNBC patients. It is consisted of two components, the active metabolite of irinotecan SN-38 and humanized RS7 antibody targeting glycoprotein Trop-2 (Marks and Kalinsky, 2017). Vahdat et al. also conducted a single-arm, multicenter trial to assess the effect of sacituzumab govitecan in patients with advanced/refractory mTNBC, administered 10 mg/kg starting dose on days 1 and 8 of 21-days repeated cycles (Bardia et al., 2017). Trop-2 was moderate to strongly positive in tumor tissue from most enrolled patients. In this cohort of heavily treated m TNBC patients, sacituzumab govitecan was shown to be very well tolerated and to produce early and long-lasting therapeutic effects.

PF-06647263, an ADC consisting of an anti-EFNA4 antibody linked to a calicheamicin payload, was shown to exhibit preliminary effects in some xenograft models (Fraguas-Sánchez et al., 2022). Ignacio et al. implemented a first-in-human study of PF-06647263 using every 3 weeks and every week regimens in patients with TNBC, ovarian cancer, and other advanced solid tumors (Garrido-Laguna et al., 2019). But as adequate exposure of PF-06647263 did not elicit enough anti-tumor activity in patients with TNBC and ovarian cancer, this study was definitively discontinued.

Mirvetuximab soravtansine is denoted as a novel ADC that has posed potential efficacy for targeting FRα-positive solid tumors (O’Malley et al., 2020). Mirvetuximab soravtansine was investigated in a prospective phase II trial for the benefit of metastatic TNBC (Yam et al., 2021). The study was terminated early due to a low rate of FRα positivity in the selected patient population and no patients had a partial or complete response. This suggested that mirvetuximab soravtansine might be an optional therapeutic ADC only when there are sufficient clinical indications or characteristic expressions.

Trastuzumab deruxtecan (T-DXD) is a conjugate of a HER2 antibody and a DNA topoisomerase I inhibitor. Trastuzumab deruxtecan has shown positive therapeutic value in a variety of tumors, such as breast cancer, gastric cancer, and non-small cell lung cancer. Several studies by Modi et al. have documented that trastuzumab deruxtecan exhibited persistent antitumor activity in a pretreated HER2-positive metastatic BC patient cohort (Tamura et al., 2019; Modi et al., 2020a). In a phase 3 trial, the percentage of patients surviving at 12 months in the trastuzumab deruxtecan group was 94.1%, among patients with previously treated HER2-positive metastatic breast cancer (Cortés et al., 2022). In another trial involving patients with HER2 low metastatic BC, Modi et al. also showed that trastuzumab deruxtecan significantly prolonged PFS and OS compared to the other chemotherapy regimens (Modi et al., 2020b; Modi et al., 2022).

In conclusion, ADC drugs can have synergistic therapeutic effects on a variety of tumors due to both the targeting and therapeutic properties of antibodies and the tumor cytotoxicity of coupled small molecule drugs. Several ADC-based therapies have been approved or are undergoing clinical trials in recent years. Although the efficacy, tolerability, and safety of ADC are the tradeoff factors affecting the efficacy of ADC, it shows a certain therapeutic prospect for TNBC tumors.

### 3.6 Others

In addition to the above, targeting EGFR in TNBC also represents a hopeful treatment option. Studies have shown that EGFR is frequently over-represented in TNBC, which suggests the potential of EGFR serving as a therapeutic target for TNBC (Carey et al., 2012). EGFR-targeted monoclonal antibodies and/or tyrosine kinase (TK) inhibitors, such as cetuximab, gefitinib, erlotinib, lapatinib, and panitumumab, allow efficient therapy for TNBC. Besides, bevacizumab is known as a world-famous monoclonal antibody targeting vascular endothelial growth factor A (VEGF-A), which is crucial for tumor growth and TNBC metastasis. For TNBC patients with BRCA1/2 mutations, adding bevacizumab after standard NAC might increase pCR (Fasching et al., 2018).

## 4 Conclusion and perspectives

At present, the above novel targeted therapeutic drugs comprise the landscape of personalized clinical treatments for TNBC, such as platinum drugs, PARP inhibitors, ICIs, AR inhibitors as well as PI3K/AKT/mTOR targeted inhibitors (Table 2). While standard anthracycline- and taxane-based chemotherapy regimens remain the classic approach to early systemic therapy, emerging information on cancer genomes has prompted molecular characterization-driven therapeutic strategies. These personalized, single, or combinational therapies based on molecular heterogeneity are currently showing positive results.

**TABLE 2:** The current targeted therapies in clinical trials in TNBC.

Specific target	Drug	Drug type	Cohort	Trial	Intervention
PARP	Olaparib	PARP inhibitor	Untreated unilateral or bilateral primary TNBC (*n* = 274)	Phase II trial NCT02789332	Olaparib + paclitaxel vs. Paclitaxel + carboplatinum
	Veliparib	PARP inhibitor	Untreated stage II-III TNBC (*n* = 634)	Phase III BrighTNess trial NCT02032277	Paclitaxel + carboplatin + veliparib vs. Paclitaxel + carboplatin + veliparib placebo vs. Paclitaxel + carboplatin placebo + veliparib placebo
	niraparib	PARP inhibitor	Advanced or metastatic TNBC	Phase II trial NCT02657889	Niraparib + pembrolizumab
	Talazoparib	PARP inhibitor	Metastatic BC with a germline BRCA1/2 mutation (*n* = 140)	Phase II trial NCT02034916	Talazoparib
	Rucaparib	PARP inhibitor	TNBC without treatment	Phase II trial EudraCT 2014–003319-12	Rucaparib
AR	Bicalutamide	AR inhibitor	ER and PR-negative metastatic BC (*n* = 424)	Phase II trial NCT00468715	Bicalutamide
	Enzalutamide	AR inhibitor	locally advanced or metastatic AR-positive TNBC (*n* = 165)	Phase II trial NCT01889238	Enzalutamide
	Abiraterone acetate	AR inhibitor	AR-positive TNBC (*n* = 146)	Phase II trial NCT01842321	Abiraterone acetate + prednisone
	Enobosarm	nonsteroidal selective AR modulator	AR-positive TNBC (*n* = 16)	Phase II trial NCT02971761	Enobosarm + pembrolizumab
PI3K/AKT/mTOR	Alpelisib	PI3Kα inhibitor	Recurrent TNBC or recurrent BC of any subtype with a germline BRCA mutation (*n* = 17)	Phase Ib trial NCT01623349	Alpelisib
	Ipatasertib	AKT inhibitor	Operable stage IA–IIIA TNBC (*n* = 151)	Phase II trial NCT02301988	Paclitaxel + ipatasertib vs. paclitaxel + placebo
	Everolimus, temsirolimus	mTORC1 inhibitor	Metaplastic TNBC (*n* = 52)	Phase 1 trial NCT00761644	Doxorubicin, bevacizumab temsirolimus or everolimus
	capivasertib	AKT inhibitor	Untreated metastatic TNBC (*n* = 140)	Phase II trial NCT02423603	Paclitaxel + capivasertib vs. Paclitaxel + Placebo
	Taselisib	PI3K inhibitor	AR-positive TNBC (*n* = 78)	Phase Ib/II study NCT02457910	Enzalutamide + taselisib
TROP2	Sacituzumab govitecan	Antibody-drug conjugate targeting TROP-2	Refractory or relapsed TNBC	Phase I/II NCT01631552	Sacituzumab govitecan
VEGF	Bevacizumab	VEGF monoclonal antibody	HER2-negative BC (*n* = 493)	Phase III trial NCT00567554	Anthracycline + taxanevs + bevacizumab vs. Anthracycline + taxanevs
	Ramucirumab	VEGFR-2 monoclonal antibody	HER2-negative, unresectable, locally recurrent, or metastatic BC (*n* = 1,144)	Phase III trial NCT00703326	Docetaxel + ramucirumab vs.Docetaxel + placebo
JAK/STAT	Ruxolitinib	JAK1/2 inhibitor	Metastatic TNBC or inflammatory BC of any subtype (*n* = 21)	Phase II trial NCT01562873	Paclitaxel + Cobimetinib vs.Paclitaxel + Placebo
Chk1	UCN-01	Multi-targeted protein kinase inhibitor	TNBC patients received up to three prior chemotherapeutic regimens (*n* = 25)	Phase II trial NCT00031681	UCN-01 + Irinotecan
EGFR	Erlotinib	EGFR inhibitor	Metastatic TNBC (*n* = 55)	Phase II trial NCT0073340	Nab-Paclitaxel + bevacizumab + bevacizumab + erlotinib
	Gefitinib	EGFR inhibitor	Women with unilateral, primary operable, TNBC ≥ 2 cm	Phase II trial NCT 00239343	Gefitinib + epirubicin + cyclophosphamide
	Lapatinib	EGFR inhibitor	Biopsy-proven, metastatic or locally advanced, unresectable TNBC (*n* = 20)	Single institution pilot trial NCT02158507	Lapatinib + veliparib
	Panitumumab	EGFR monoclonal antibody	TNBC with no prior surgery/chemo/hormonal/anti-EGFR and radiation therapy (*n* = 60)	Neoadjuvant phase II trial NCT00933517	Panitumumab + 5-fluorouracil + epidoxorubicin + cyclophosphamide + docetaxel
	Cetuximab	EGFR monoclonal antibody	metastatic TNBC (*n* = 102)	Phase II trial NCT00232505	Cetuximab + carboplatin

Although the current studies have all made some research progress, they are still challenging at this time. Firstly, it must be emphasized that TNBC is a highly heterogeneous tumor disease, including many different entities with different biological and clinical behaviors. The molecular characteristics of TNBC are not only important indicators for tumor evaluation, but also important references for the “tailored” targeted therapy and diagnosis of TNBC. Increasingly updated multiomics studies, including genomic, transcriptome, metabolome and epigenetic, provide detailed information on the deeper characterization of TNBC. There is a need to establish specific molecular profiles or metrics to precisely evaluate treatment benefits and risks and to determine better trial design options. Integrating multiple strategies such as tumor proliferation/immune-related markers and PAM50 subtypes allows adjustment for further step-down chemotherapy and/or other therapies in treating TNBC. Secondly, the cohorts included in some studies contain small numbers of patients and these patients are from a single institution. In addition, for some combination therapies, the optimal dose ratios or sequential dosing regimens need to be further explored to determine the best combination patterns, for optimal effects. Thus, at present, the main focus of existing studies is on the ongoing and completed early trials, the efficacy outcomes and common adverse events of each type of treatment, and to guide the adjustment and protocol involved in subsequent drug therapy Multicenter, long-follow, large-cohort clinical treatment investigation are still necessary to determine exact efficacy of these drugs.

Precision oncology, by analyzing the transcriptome, proteome and metabolome of the tumor genome in order to identify targets that can be sought to attack the tumor and develop treatment strategies accordingly. Classification of specific molecular profiles of TNBC to provide diagnosis, prognosis, and treatment information has been applied in clinical trails for choosing targeted therapies such as small molecule inhibitors or monoclonal antibodies and for predicting therapeutic resistance. Since tumors have a huge number of molecular alterations, it may be arduous to achieve clinical efficacy by targeting just one or several of the mutation. There is no denying that precision oncology does bring a ray of hope to the treatment of TNBC. Collectively, based on TNBC molecular classification, it is currently necessary to mine therapeutic targets within each subtype and formulate corresponding therapeutic strategies. Future research still needs to develop highly effective targeted drugs and identify relevant biomarkers to evaluate therapeutic benefits.

## Glossary

ADCs: antibody-drug conjugates
AR: androgen receptor
LAR: luminal androgen receptor: luminal androgen receptor
BLIA: basal-like immune-activated
BLIS: basal-like immunosuppressed
BL: basal-like subtypes
BC: Breast cancer
CNVs: copy number variants
ctDNA: circulating tumor DNA
CL: claudin-low
EGFR: epidermal growth factor receptor
EMT: epithelial-to-mesenchymal transition
ER: estrogen receptor
CDX-011, GV: glembatumumab vedotin
HR: hormone receptor
HRD: homologous recombination defect
HER2: human epidermal growth factor receptor 2
ICIs: immune checkpoint inhibitors
IRGs: immune-related gene
IM: immunomodulatory subtypes
ITT: intention to treat
iDFS: invasive disease-free survival
LAR: luminal androgen receptor: luminal androgen receptor
MES: mesenchymal
MMAE: monomethyl auristatin E
MSL: mesenchymal stem-like subtypes
MpBC: metaplastic breast cancers
mTNBC: metastatic triple-negative breast cancer
ORR: objective response rate
OPN: osteopontin
OS: overall survival
PCb: paclitaxel plus carboplatin
pCR: pathological complete response
PI3K: phosphoinositide-3 kinase
PBC: platinum-based chemotherapy
PARP: poly ADP-ribose polymerase
PR: progesterone receptor
PD-L1: programmed cell death ligand 1
PFS: progression-free survival
RFS: recurrence-free survival
SARM: selective androgen receptor modulator
T-DXD: Trastuzumab deruxtecan
TNBC: triple-negative breast cancer
TILs: tumor-infiltrating lymphocytes
TK: tyrosine kinase
